# Correction: Maynard et al. Notes from the 2022 Folate, Vitamin B12, and One-Carbon Metabolism Conference. *Metabolites* 2023, *13*, 486

**DOI:** 10.3390/metabo13060752

**Published:** 2023-06-14

**Authors:** Adam G. Maynard, Boryana Petrova, Naama Kanarek

**Affiliations:** 1 Department of Pathology, Boston Children’s Hospital, Boston, MA 02115, USA; 2 Graduate Program in Biological and Biomedical Sciences, Harvard Medical School, Boston, MA 02115, USA; 3 Harvard Medical School, Boston, MA 02115, USA; 4 Broad Institute of Harvard and MIT, Cambridge, MA 02142, USA

## Text Correction

Thanks to feedback from several speakers, text was amended, and citations updated, in the original article [1]. A correction has been made to the following sections:

## 2. Basic Biochemistry of 1C Metabolism

Text not approved by speakers was removed. Ref. [6] was updated.

## 3. Whole-Body Folate Homeostasis

Text not approved by speakers was removed.

## 5. One-Carbon Metabolism and Cancer

### 5.1. Dependency of Cancer Cells on Methionine and the Methylation Cycle

Affiliation was added to Dr. Zhenxun Wang; Duke-National University of Singapore Medical School. Text for Caitlin Zacharias was updated to align with speaker’s consent.

### 5.2. B Vitamins and Their Role in Cancer Progression

Text for Dr. Lingbo Zhang was edited to align with speaker’s consent.

## 7. One-Carbon Metabolism and Neuronal Biology

### 7.1. One-Carbon Metabolism during Development

Text and reference for Dr. Nicholas Greene and Dr. Charles Venditti was updated to align with speakers’ consent. Additionally, some text was removed to align with speaker’s consent.

### 7.2. Postnatal Neuronal Defects Related to B12 Deficiency

Text from D. Jessica Tanis was edited to align with speakers consent. Additionally, some text was removed to align with speaker’s consent.

The authors state that the scientific conclusions are unaffected. This correction was approved by the Academic Editor. The original publication has also been updated.
